# Role of Iron-Containing Alcohol Dehydrogenases in *Acinetobacter baumannii* ATCC 19606 Stress Resistance and Virulence

**DOI:** 10.3390/ijms22189921

**Published:** 2021-09-14

**Authors:** Guang-Huey Lin, Ming-Chuan Hsieh, Hung-Yu Shu

**Affiliations:** 1 Master Program of Microbiology and Immunology, School of Medicine, Tzu Chi University, Hualien 97004, Taiwan; veronica@gms.tcu.edu.tw (G.-H.L.); 105329104@gms.tcu.edu.tw (M.-C.H.); 2 Department of Microbiology, School of Medicine, Tzu Chi University, Hualien 97004, Taiwan; 3 International College, Tzu Chi University, Hualien 97004, Taiwan; 4 Department of Bioscience Technology, Chang Jung Christian University, Tainan 71101, Taiwan

**Keywords:** iron-containing alcohol dehydrogenase, alcohol metabolism, stress resistance, virulence

## Abstract

Most bacteria possess alcohol dehydrogenase (ADH) genes (*Adh* genes) to mitigate alcohol toxicity, but these genes have functions beyond alcohol degradation. Previous research has shown that ADH can modulate quorum sensing in *Acinetobacter baumannii*, a rising opportunistic pathogen. However, the number and nature of *Adh* genes in *A. baumannii* have not yet been fully characterized. We identified seven alcohol dehydrogenases (NAD^+^-ADHs) from *A. baumannii* ATCC 19606, and examined the roles of three iron-containing ADHs, ADH3, ADH4, and ADH6. Marker-less mutation was used to generate *Adh3*, *Adh4*, and *Adh6* single, double, and triple mutants. Disrupted *Adh4* mutants failed to grow in ethanol-, 1-butanol-, or 1-propanol-containing mediums, and recombinant ADH4 exhibited strongest activity against ethanol. Stress resistance assays with inorganic and organic hydroperoxides showed that *Adh3* and *Adh6* were key to oxidative stress resistance. Virulence assays performed on the *Galleria mellonella* model organism revealed that *Adh4* mutants had comparable virulence to wild-type, while *Adh3* and *Adh6* mutants had reduced virulence. The results suggest that ADH4 is primarily involved in alcohol metabolism, while ADH3 and ADH6 are key to stress resistance and virulence. Further investigation into the roles of other ADHs in *A. baumannii* is warranted.

## 1. Introduction

Alcohol dehydrogenases (ADHs; EC 1.1.1.1 and EC 1.1.1.2) are widely present in most bacteria [1] and can mitigate the cytotoxicity of aliphatic alcohols such as ethanol, propanol, or 1-butanol [2]. ADHs can be classified by coenzyme into three main types: nicotinamide adenine dinucleotide (NAD^+^)-dependent, NAD^+^-independent, and flavin adenine dinucleotide (FAD^+^)-dependent. The NAD^+^-dependent ADHs (NAD^+^-ADHs) can be further subdivided into zinc-containing, iron-containing, and short chain ADHs [3,4,5,6]. Interestingly, not all bacteria with ADHs have ethanol-degrading capabilities [7,8,9], and previous research has shown that bacterial ADHs may have important functions beyond alcohol metabolism, being involved in such diverse mechanisms as stress resistance [10], biofilm formation [11], and bacterial virulence [12].

In addition, several studies have shown that the presence of ethanol can induce stress responses in bacteria that lead to increased virulence [13,14,15], and this has particularly been noted for *Acinetobacter baumannii*, a rising opportunistic pathogen that is increasingly seen in nosocomial and community-acquired infections [14,15]. In the presence of ethanol, *A. baumannii* upregulated the expression of several proteins involved in stress responses, iron assimilation, phosphate transport, and lipid and carbohydrate anabolism; while at the same time, increased secretion of phospholipase C, acidification of bacterial cultures, and formation of biofilms was also noted [14,15]. The main *A. baumannii Adh* gene (*A1S_2098*) was strongly induced in the presence of ethanol, but other genes potentially encoding ADHs, including two iron-containing ADHs (FeADHs), were not induced or only slightly upregulated [14], and this suggests that these ADHs may have functions beyond ethanol degradation. Importantly, it was found that ethanol-exposed *A. baumannii* demonstrated stronger virulence in a *Galleria mellonella* model of infection [15]. A recent study has also shown that the *A1S_2098* gene is significantly upregulated during the *A. baumannii* biofilm formation process, and may play a role in quorum sensing, bacterial growth, and motility [11]. However, the number and nature of other *A. baumannii* genes potentially encoding ADHs, particularly FeADHs, has not yet been explored in detail.

In this study, we sought to elucidate the roles of three *A. baumannii* genes identified through in silico methods as potentially encoding FeADHs, and assessed the impact of their expression products on ethanol metabolism, stress responses, and virulence. These results can help to bridge the gaps in our current understanding of ADH function in *A. baumannii*, and may serve as the basis for future research into other as yet uncharacterized *Adh* genes. In the long term, the results of such research could potentially advise more effective sanitizing procedures and infection control measures, in order to prevent the survival and spread of *A. baumannii* in both clinical and community settings.

## 2. Results

### 2.1. In Silico Identification of Putative ADH Genes in A. baumannii

A search of the GenBank database revealed seven genes annotated as possible *Adh* genes in *A*. *baumannii* (Table S1). Genomic analysis revealed that three of these are iron-containing ADHs (ADH3, ADH4, and ADH6), another three are zinc-dependent ADHs (ADH1, ADH2, and ADH7), and the remaining one is a short-chain ADH (ADH5). The iron-containing ADHs were respectively encoded by *DJ41_189* (ADH3; 1,158 bp), *DJ41_136* (ADH4; 1,173 bp), and *DJ41_1604* (ADH6; 1,185 bp). Interestingly, the number of annotated *Adh* genes is relatively low in terms of the genome size of *A. baumannii* (genome size 3.97 Mb, 7 *Adh* genes annotated in GenBank), as other Gram-negative soil bacteria that are also opportunistic pathogens have significantly more annotated *Adh* genes, with 44 *Adh* genes found for *Burkholderia psedomallei* K96243 (7.42 Mb genome) and 42 *Adh* genes annotated for *Pseudomonas aeruginosa* PAO1 (6.26 Mb genome). However, this may be indicative of limited research conducted in *A. baumannii*, and further studies are needed to better examine this phenomenon.

**Figure 1:**
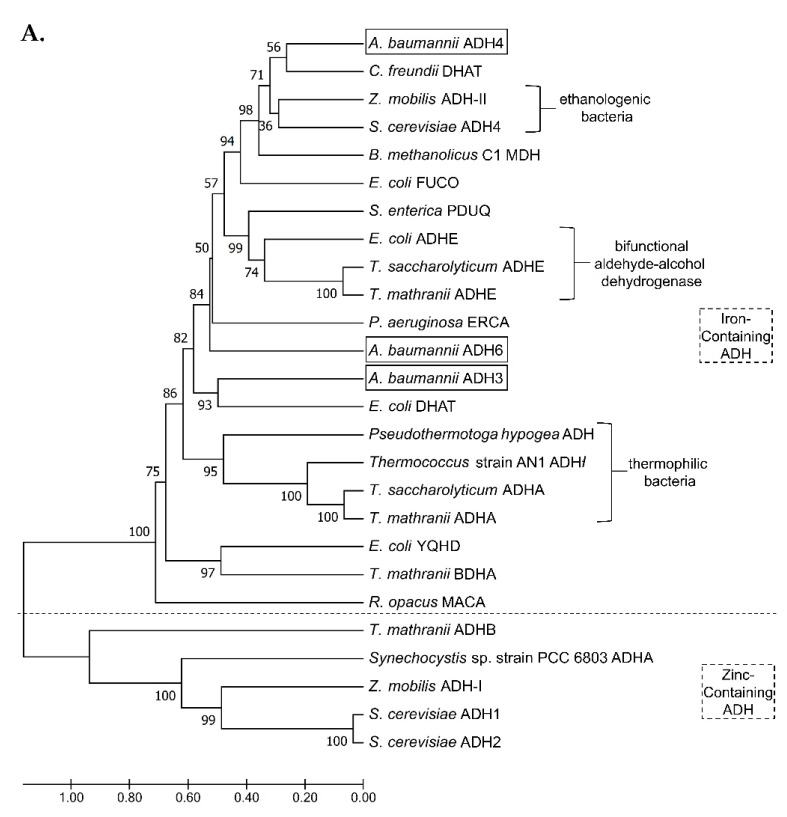

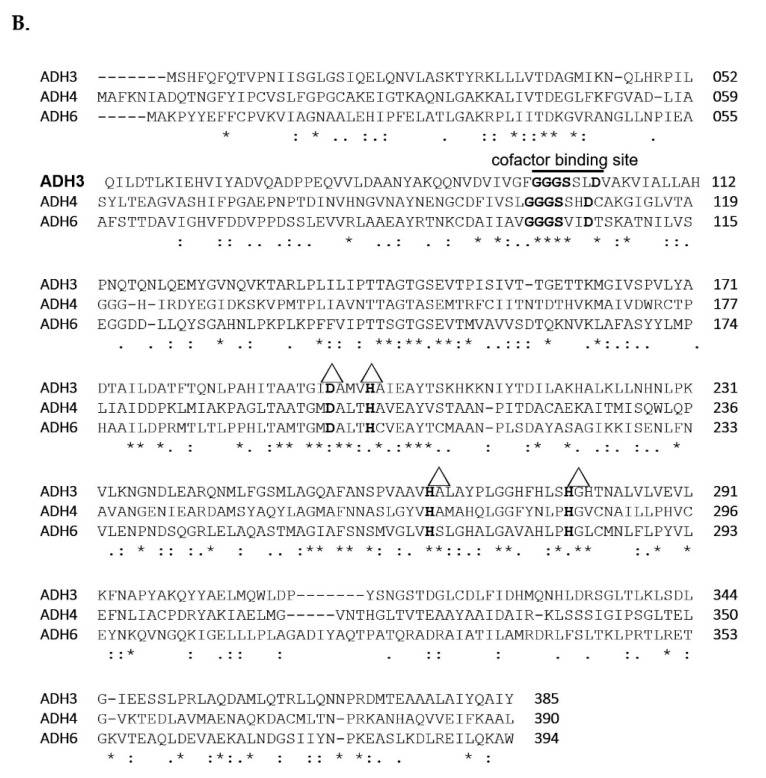
(**A**) Cladogram of iron-containing *Adh* genes in *A. baumannii* compared with 23 other *Adh* genes from 14 organisms. The scale bar indicates the number of nucleotide substitutions per site. (**B**) Amino acid alignment of three FeADHs (ADH3, ADH4, ADH6) in *A. baumannii*. Δ indicates the positions of the four key iron-binding sites (D, H, H, H). Asterisks (*) indicate positions with single fully conserved amino acid residues; colons (:) indicate positions with conservation between residues of strongly similar properties (scoring > 0.5 in the Gonnet point accepted mutation 250 matrix); and periods (.) indicate positions with conservation between groups of weakly similar properties (scoring ≤ 0.5 in the Gonnet point accepted mutation 250 matrix).

Phylogenetic analysis was conducted with 26 annotated ADH amino acid sequences from 14 microorganisms that were identified in a comprehensive search of the GenBank database, and MEGA 7 software (https://www.megasoftware.net/, accessed on 31 July 2021) was used to construct a phylogenetic tree. The resulting dendrogram revealed two discrete iron-containing and zinc-containing clades (Figure 1). Of the iron-containing ADHs identified in *A. baumannii*, ADH4 was found to distribute in the same clade as ADH from ethanologenic microorganisms, including *Saccharomyces cerevisiae* (Figure 1A). ADH3 and ADH6 shared higher identity with bifunctional aldehyde-alcohol dehydrogenases (Figure 1A). Proteomic analysis showed that ADH3 shared 31.7% identity with ADH4, and 30.6% identity with ADH6, while ADH4 and ADH6 shared 33.1% identity (Figure 1B). The cofactor binding motif, GGGSXXD, four key iron-binding sites (D, H, H, H), and a highly conserved aspartic acid residue (D; located at position 39 in ADH3, position 46 in ADH4, and position 41 in ADH6) were found in all three FeADHs, and indicate the preference for NAD+ as a cofactor for these enzymes (Figure 1B).

### 2.2. Biochemical Properties of ADH4

To understand the biochemical properties of these three FeADHs, we cloned their encoding genes into plasmid pQE80L, then transformed plasmids into *E. coli* for protein overexpression and subsequent purification. Overexpression was successfully induced for all FeADHs (Figure S1, lanes 2, 5, 8), and soluble proteins were purified by Ni-affinity chromatography, with protein sizes of about 43.7 kDa, 44.2 kDa and 44.7 kDa, respectively (Figure S1, lanes 3, 6, 9).

**Figure 2:**
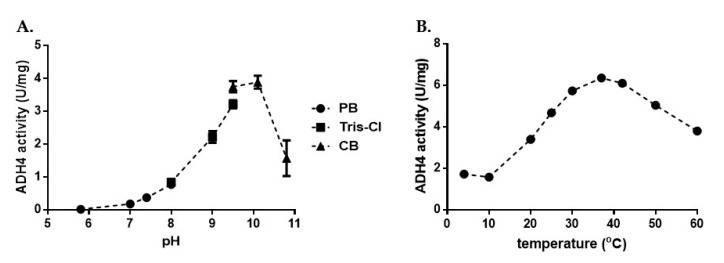
(**A**) Optimum pH conditions for ADH4 enzymatic activity. ADH4 enzymatic activity was assessed at room temperature under the following pH ranges: Tris-Cl: pH = 5.8–8; PB, phosphate buffer: pH = 8–9.5; CB, carbonate-bicarbonate buffer: pH = 9.5–10.8. The production of 1 µmole of NADH was defined as 1 unit of ADH activity. (**B**) Optimum temperature of ADH4 enzymatic activity. ADH4 enzymatic activity was assessed for different temperatures at the optimum pH of 10.8 in CB buffer.

The enzymatic activity of ADH4 was tested in different buffers, as well as varying temperature conditions, in order to establish the optimal conditions for enzymatic activity assays against different alcohol substrates. We tested 0.5 µM of ADH4 in 80 µM of three different buffers (Figure 2A), and the highest activity was noted with the CB buffer at a pH value of 10.1 (Figure 2A). In addition, maximal ADH4 activity was detected at 37 °C (Figure 2B). Using these conditions, we proceeded to assess the substrate specificity of recombinant ADH4 against ethanol, 1-propanol, and 1-butanol, and subsequently found that the Michaelis–Menten constant (*K_M_*) was lowest for ethanol (Table 1; Figure S2), indicative of stronger affinity to ethanol for ADH4 over other aliphatic alcohols.

### 2.3. Biological Function of ADH3, ADH4, and ADH6

**Figure 3:**
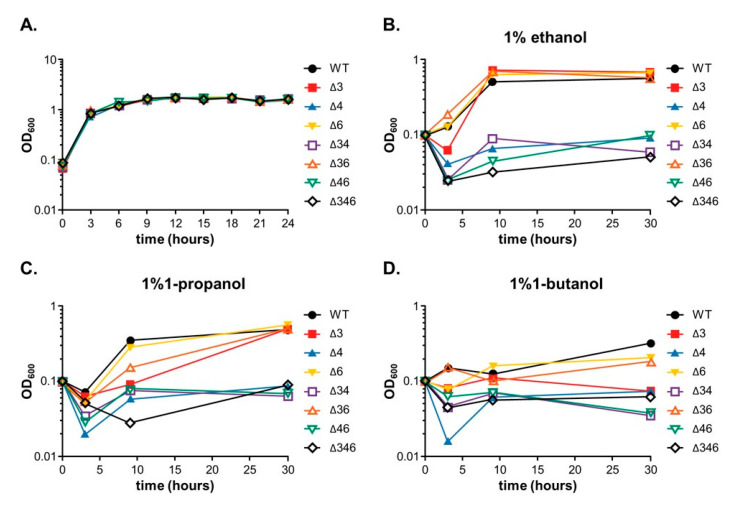
Growth rates of wild-type (WT) or mutant strains in different medium. (**A**) Growth rates in LB medium. (**B**) Growth rates in M9 medium containing 1% ethanol. (**C**) Growth rates in M9 medium containing 1% 1-propanol. (**D**) Growth rates in M9 medium containing 1% 1-butanol.

To elucidate the biological roles of *Adh3, Adh4* and *Adh6*, we generated three single mutants (Δ*3*, Δ*4*, Δ*6*), three double mutants (Δ*34*, Δ*36*, Δ*46*), and one triple mutant (Δ*346*) using marker-less mutation. Mutants were confirmed by colony PCR (Figures S3 and S4) with gene-specific primers (Table 2). Growth rates of these seven mutants in LB medium did not differ from wild-type, suggesting that FeADHs did not play a major role in growth and survival under culturing conditions with rich medium (Figure 3A). However, strains with mutated *Adh4* failed to proliferate in M9 medium containing 1% ethanol (Figure 3B), in line with the findings in Table 1 that point to a role for ADH4 in ethanol metabolism. Moreover, all strains with mutated *Adh4* failed to proliferate in medium containing 1% of 1-propanol, while Δ*3* and Δ*36* mutants experienced reduced growth rates compared to wild-type (Figure 3C). Interestingly, the Δ*6* mutant had comparable growth rates as wild-type, suggesting that both ADH3 and ADH4 may have roles in 1-propanol metabolism. All mutant strains had reduced growth compared to wild-type in medium containing 1% of 1-butanol (Figure 3D).

### 2.4. Gene Expression Patterns of Wild-Type and Mutant Strains in the Presence of Ethanol

**Figure 4:**
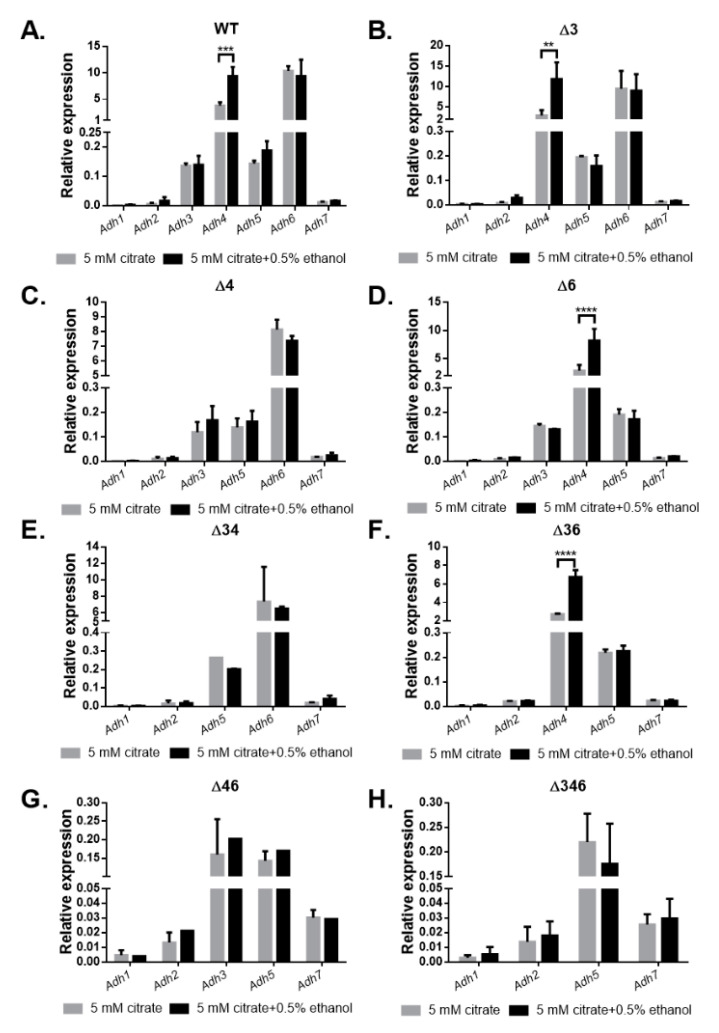
Relative gene expression of *Adh* genes in wild-type and mutant strains after culturing in medium with or without ethanol. The *gyrase* gene (*A4U85_RS04125*) was used as a control to calculate relative expression levels. Comparison of relative gene expression for *A. baumannii* ATCC 19606 (**A**) wild-type; (**B**) Δ*3* single mutant; (**C**) Δ*4* single mutant; (**D**) Δ*6* single mutant; (**E**) Δ*34* double mutant; (**F**) Δ*36* double mutant; (**G**) Δ*46* double mutant; and (**H**) Δ*346* triple mutant strains cultured in M9 medium with 5 mM citrate (gray bar) or 5 mM citrate and 0.5% ethanol (black bar). Two-way ANOVA tests were conducted to assess statistical significance. ** *p* < 0.01; *** *p* < 0.001; **** *p* < 0.0001.

Gene expression patterns of wild-type and mutant strains cultivated in 5 mM citrate medium or 5 mM citrate medium with 0.5% ethanol were compared through transcript analysis, with the *gyrase* gene (*A4U85_RS04125*) as a control to calculate relative expression levels (Figure 4). In the wild-type strain, it can be seen that genes encoding FeADHs were more highly expressed than the other *Adh* genes, with the exception of *Adh5* (Figure 4A). Expression levels of *Adh*3 and *Adh*6 did not change in the presence of ethanol, but *Adh4* expression significantly increased by 3.79- to 9.39-fold (*p* < 0.001) in the ethanol-containing medium (Figure 4A). In mutant strains, genes encoding FeADHs were also more highly expressed than the other *Adh* genes, with the exception of *Adh5* (Figure 4B–H). In the Δ*3* (Figure 4B), Δ*6* (Figure 4D), and Δ*36* (Figure 4F) mutant strains, *Adh4* expression also significantly increased in the presence of ethanol (*p* < 0.01). These results suggest that ADH4 plays a significant role in ethanol metabolism, while ADH3, ADH5, and ADH6 may have other key functions that necessitate a high level of expression, regardless of the presence of ethanol.

### 2.5. Adh3 and Adh6 Are Involved in Inorganic and Organic Oxidative Stress Responses

**Figure 5:**
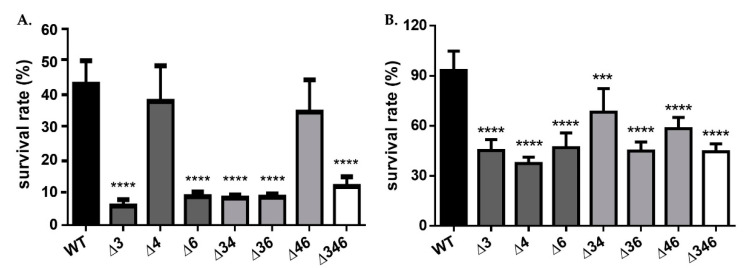
Survival rates of wild-type or mutant strains treated with (**A**) 5 mM H_2_O_2_; or (**B**) 300 mM *tert*-BHP for 20 min. Two-way ANOVA tests were conducted to assess statistical significance. *** *p* < 0.001; **** *p* < 0.0001.

Previous research has suggested that AdhA in *Synechocystis* sp. PCC 6803 may help to increase ethanol tolerance by maintaining an adequate level of NADH or NADPH to counteract oxidative stress [16], and thus we considered the possibility that FeADHs may similarly act to counterbalance oxidative stress by maintaining the homeostasis of NADH or NADPH in *A. baumannii* cells. We accordingly cultured wild-type and mutant strains in LB medium with an initial OD_600_ of 0.1. Viable bacterial counts were determined when the OD_600_ reached 0.6, after which cultures were treated with 5 mM H_2_O_2_ for 20 min. Viable bacterial counts were again assessed, and survival rates were then determined by comparing with pre-testing viability counts (Figure 5). The results showed that only the Δ*4* single mutant and the Δ*46* double mutant maintained comparable survival rates after H_2_O_2_ treatment (Figure 5A), indicating a critical role for ADH3 in countering inorganic oxidative stress. The same experiment was performed again, but 300 µM of organic *tert*-BHP was applied for 20 min instead of H_2_O_2_. The results showed that all mutants experienced significant reductions of at least 30% in viability as compared to wild-type, and this suggests that all three FeADHs may contribute to organic hydroperoxide resistance (Figure 5B).

**Figure 6:**
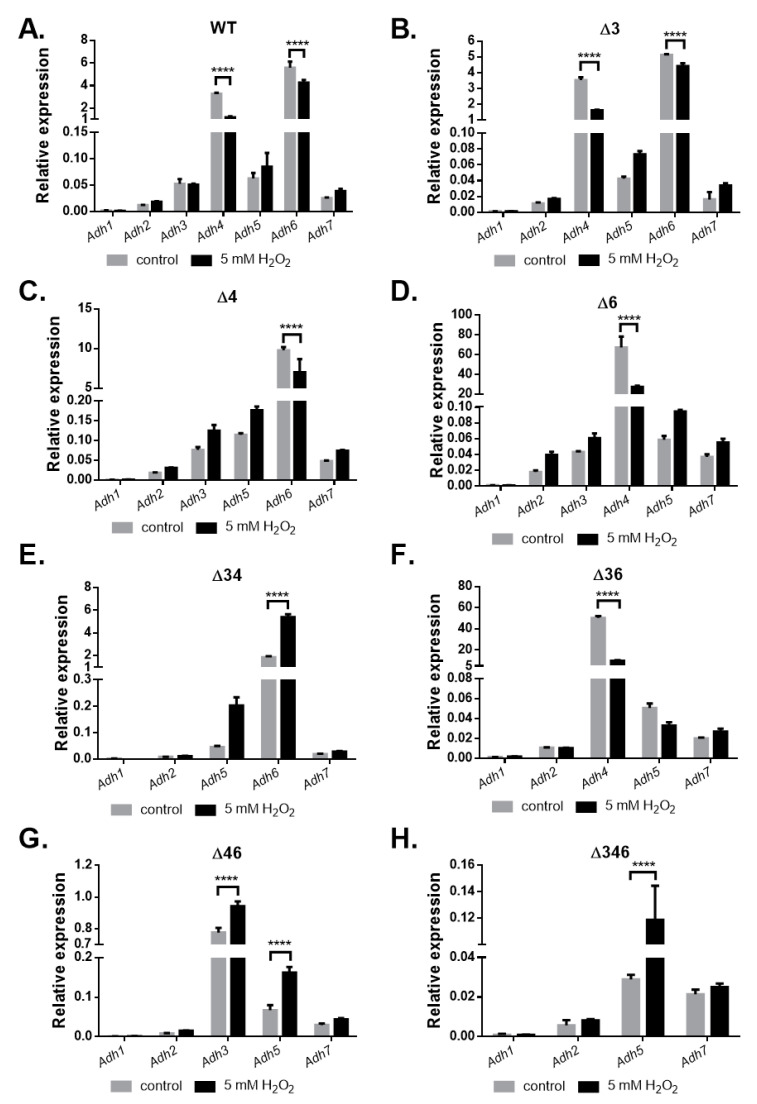
Relative gene expression of *Adh* genes in wild-type and mutant strains after treatment with H_2_O_2_ for 20 min. Comparison of relative gene expression for *A. baumannii* ATCC 19606 (**A**) wild-type; (**B**) Δ*3* single mutant; (**C**) Δ*4* single mutant; (**D**) Δ*6* single mutant; (**E**) Δ*34* double mutant; (**F**) Δ*36* double mutant; (**G**) Δ*46* double mutant; and (**H**) Δ*346* triple mutant strains cultured in M9 medium before (gray bar) and after 5 mM H_2_O_2_ treatment for 20 min (black bar). Two-way ANOVA tests were conducted to assess statistical significance. **** *p* < 0.0001.

**Figure 7:**
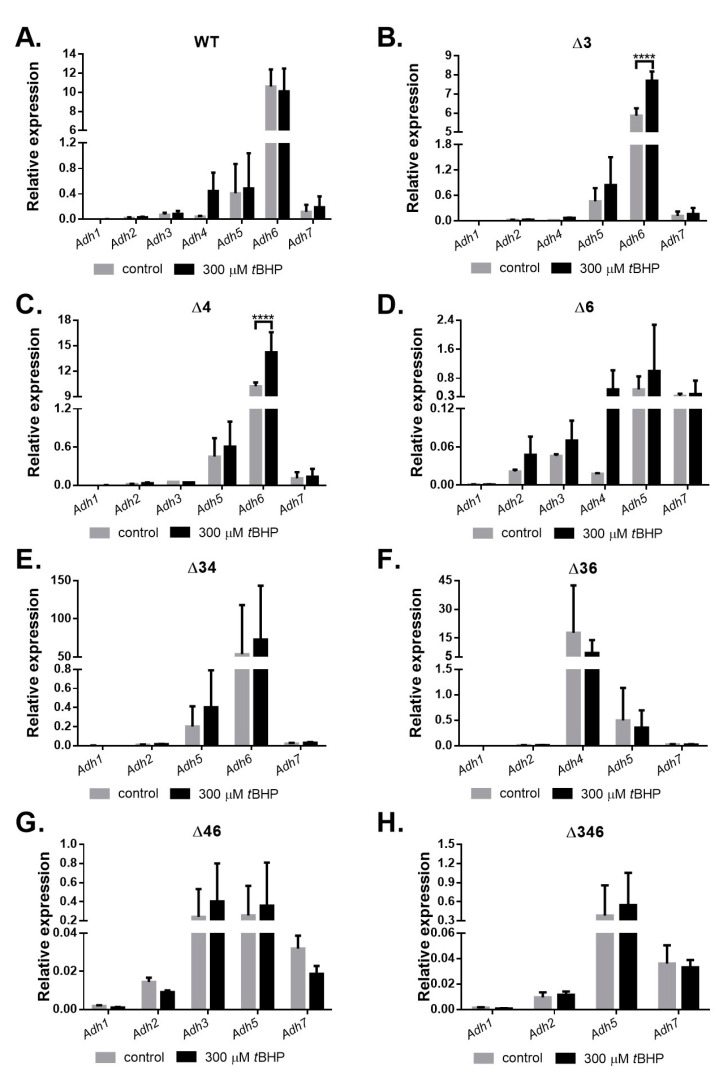
Relative gene expression of *Adh* genes in wild-type and mutant strains after treatment with *tert*-BHP for 20 min. Comparison of relative gene expression for *A. baumannii* ATCC 19606 (**A**) wild-type; (**B**) Δ*3* single mutant; (**C**) Δ*4* single mutant; (**D**) Δ*6* single mutant; (**E**) Δ*34* double mutant; (**F**) Δ*36* double mutant; (**G**) Δ*46* double mutant; and (**H**) Δ*346* triple mutant strains cultured in M9 medium before (gray bar) and after 300 mM *tert*-BHP treatment for 20 min (black bar). Two-way ANOVA tests were conducted to assess statistical significance. **** *p* < 0.0001.

Cultures were collected for RNA extraction, and differential gene expression under inorganic and organic oxidative stress was analyzed using qRT-PCR. The results showed that no *Adh* genes were upregulated in the wild-type strain after H_2_O_2_ treatment; in fact, expression levels of *Adh4* and *Adh6* decreased significantly (Figure 6A). However, after H_2_O_2_ treatment, *Adh6* expression increased significantly in the Δ*34* double mutant strain (Figure 6E); *Adh3* and *Adh5* expression increased significantly in the Δ*46* double mutant strain (Figure 6G), and *Adh5* expression increased significantly in the Δ*346* triple mutant strain (Figure 6H). These findings suggest that *Adh5* may partially complement inorganic oxidative stress responses when *Adh3* and *Adh6* are not available. Following *tert*-BHP treatment, no *Adh* genes were upregulated in the wild-type strain (Figure 7A), but *Adh6* expression increased significantly in Δ*3* single mutant (Figure 7B) and Δ*4* single mutant (Figure 7C) strains. *Adh3* expression increased significantly in the Δ*46* double mutant (Figure 7G), while *Adh5* expression also significantly rose in both the Δ*46* double mutant (Figure 7G) and Δ*346* triple mutant strains (Figure 7H), presumably to complement the stress response in the absence of *Adh6* and *Adh3*. Taken together, these results point to a critical role for *Adh3* and *Adh6* in countering inorganic and organic oxidative stress.

### 2.6. The Adh3 Stress Resistance Response Does Not Disrupt Homeostasis of Cytosolic NADH/NAD^+^

**Figure 8:**
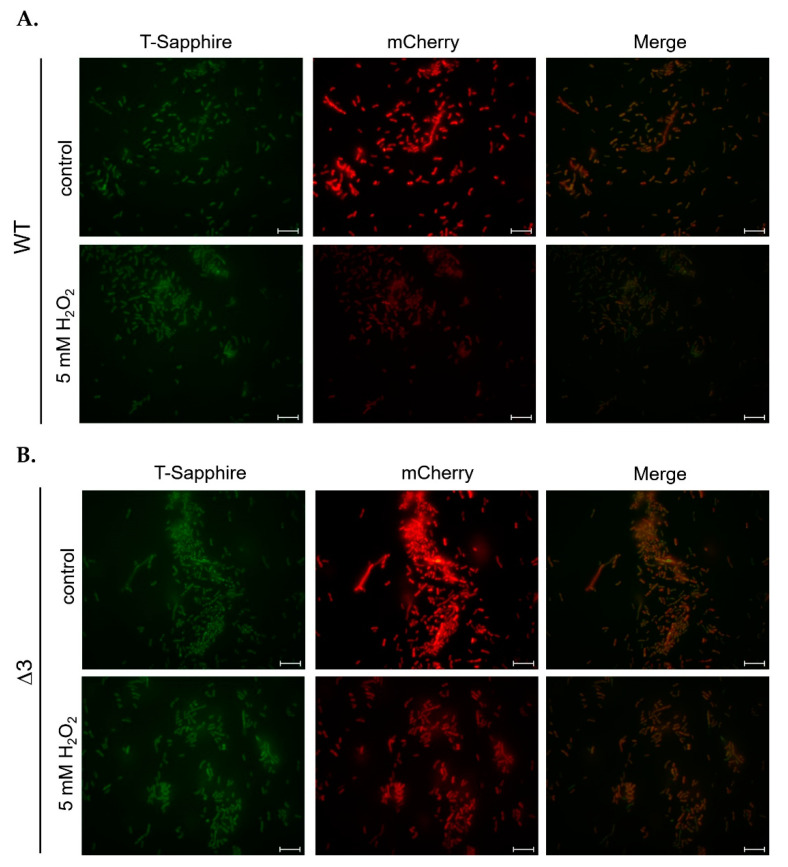
Fluorescence analysis of *A. baumannii* ATCC 19606 (**A**) wild-type and (**B**) Δ*3* single mutant with a Peredox plasmid. Here, mCherry fluorescence reflects bacterial viability, while T-Sapphire fluorescence reflects redox changes in bacteria. A total of 5 µL of bacterial culture before (control) and after 5 mM H_2_O_2_ treatment for 20 min were added to slides before observation, with mCherry images captured after 4 s of excitation at a wavelength of 587 nm, while T-Sapphire images were captured after 8 s of excitation at a wavelength of 400 nm. Scale bars: 10 µm.

To ascertain if any ADHs were involved in NADH/NAD^+^ homeostasis, the plasmid pWH1266-peredox-mCherry, containing a Peredox cassette, was transformed into wild-type and mutant strains for qualitative and quantitative fluorescence analysis. When transformed strains were treated with 5 mM H_2_O_2_ for 20 min, the intensity of mCherry fluorescence was reduced in both wild-type (Figure 8A) and Δ*3* single mutant (Figure 8B) strains, indicative of reduced bacterial viability. However, the intensity of T-Sapphire fluorescence in both strains was unaffected by H_2_O_2_ treatment (Figure 8), and this indicates that the oxidative stress response mediated by ADHs does not impact the homeostasis of cytosolic NADH/ NAD^+^.

### 2.7. Adh3 and Adh6 Are Associated with Virulence against G. mellonella

**Figure 9:**
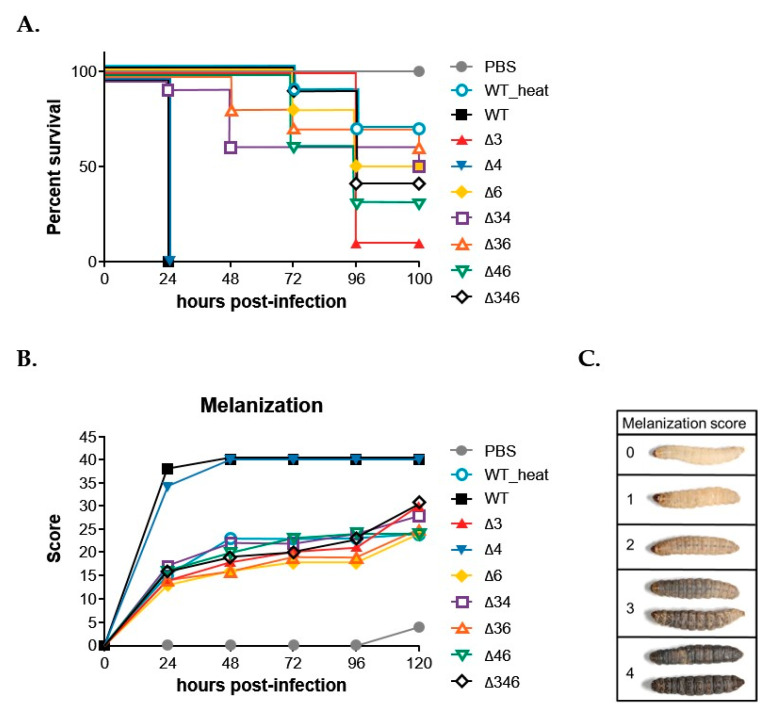
*G. mellonella* survival rates and melanization scores after infection with *A. baumannii* ATCC 19606 wild-type (WT) and mutant strains. (**A**) Kaplan–Meier survival curves, with each curve representing a single experiment performed with 10 larvae. (**B**) Melanization score curves. (**C**) Pictorial definition of melanization scores for reference. Larvae were infected with 5 × 10^6^ CFU of wild-type or mutant strains, with PBS used as the buffer and serving as a control. WT_heat indicates wild-type *A. baumannii* treated at 100 °C for 10 min.

Correlations between higher stress resistance and higher virulence have previously been reported for *A. baumannii* [17], and we sought to ascertain if ADHs were involved in virulence, using a common model organism, *G. mellonella* [15,18,19]. We injected 5 × 10^6^ CFU of wild-type and mutant strains into sets of 10 *G. mellonella* larvae, with PBS and heat-treated *A. baumannii* serving as controls (Figure 9). Kaplan–Meier survival curves showed that all 10 larvae injected with wild-type or Δ*4* single mutant strains died within 24 h of injection, while all 10 PBS-injected larvae remained alive at 120 h post-injection (Figure 9A). Larvae injected with heat-treated *A. baumannii* experienced 70% less mortality at 120 h post-injection, and reduced mortality was also observed for the Δ*36* double mutant, Δ*34* double mutant, and Δ*6* single mutant strains, which maintained >50% survival rates at 120 h post-injection (Figure 9A). Larvae injected with the Δ*346* triple mutant strain also maintained a 40% survival rate at 120 h post-injection (Figure 9A). These results suggest that *Adh3* and *Adh6* likely contribute to virulence against *G. mellonella*. We also examined the melanization of *G. mellonella* larvae after injection, as this process is a key part of the insect response to bacterial infection, with melanin aggregation observable around microbes within the hemolymph. This is believed to promote pathogen killing. Melanization status was scored daily as shown in Figure 9C, with larvae that are white in color throughout the body scored as 0, those with dark spots on the head and tail scored as 1, those with dark lines appearing on the body scored as 2, those with dark lines across the entire body scored as 3, and those that appear fully darkened scored as 4. The highest melanization scores were observed in larvae injected with wild-type or Δ*4* single mutant strains, in accordance with the survival results. However, larvae injected with other mutant strains exhibited similar levels of melanization as heat-treated *A. baumannii* (Figure 9B).

## 3. Discussion

In this study, we identified seven annotated *Adh* genes in *A. baumannii* ATCC 19606 following a search of GenBank, and further examined the roles of three FeADHs: ADH3, ADH4, and ADH6. We found that ADH4 played a key role in ethanol metabolism, as observed from substrate specificity assays (Table 1), viability assays in alcohol-containing medium (Figure 3), and gene expression patterns in the presence of ethanol (Figure 4); while ADH3 and ADH6 were mainly involved in the oxidative stress response and were associated with virulence, as seen in the results of oxidative stress assays (Figure 5), gene expression patterns in the presence of inorganic and organic hydroperoxides (Figure 6 and Figure 7), and *G. mellonella* virulence assays (Figure 9). The results suggest that ADHs may play a broad role in *A. baumannii* beyond ethanol metabolism, and regarding issues such as the potential role of ADH5 in complementing the oxidative stress response in the absence of ADH3 and ADH6 expression, the relatively low number of *Adh* genes annotated thus far in terms of the genome size of *A. baumannii*, and the roles of other non-iron-containing ADHs, further research is warranted.

Genomic and proteomic analysis of the three FeADHs examined in this study showed that the GGGS cofactor binding site motif (GGGSXXD) was conserved in all FeADHs [1,8], and four key iron-binding sites (D, H, H, H) and a key aspartic acid residue (D; located at position 39 in ADH3, position 46 in ADH4, and position 41 in ADH6) were also found in all three FeADHs (Figure 1B). The aspartic acid residue shows a preference for NAD^+^ as the cofactor, as this residue is replaced with glycine (G) in some FeADHs in other microorganisms [20,21], resulting in a shift to NADP^+^ as the preferred cofactor [22,23]. It has been suggested that the shorter side chain of glycine allows more space for the phosphate of NADP^+^ [1], while aspartic acid has a longer side chain that only allows space for NAD^+^ binding [24].

We found that *A. baumannii* was able to survive in medium containing only ethanol, 1-propanol, or 1-butanol as the sole energy source (Figure 3), and although ADH4 had higher affinity for ethanol (Table 1), enzymatic activity against 1-propanol and 1-butanol was also detected. This is consistent with findings for ADHs in other microorganisms, which typically have activity against most aliphatic alcohols [23,25,26], although some substrates may be preferred over others; however, there are also ADHs that have strong specificity for a particular alcohol [27,28]. A diverse range of enzymatic activity enables *A. baumannii* to survive under challenging conditions, make use of various energy sources, and demonstrate greater stress resilience overall.

Oxidative stress assays (Figure 5) and gene expression patterns in the presence of inorganic (Figure 6) and organic (Figure 7) hydroperoxides indicate that ADH3 and ADH6 have critical roles in the oxidative stress response, and this has been observed for ADHs in other microorganisms as well [10,16,29], some of which are also involved in the stress response to heat and other stressors [10,29]. ADHs have also been reported to be involved in bacterial biofilm formation [11], and *Adh* expression was shown to be upregulated in *A. baumannii* ATCC 17978 strains grown under biofilm conditions [11]. Biofilm formation can increase stress tolerance, but the exact role of ADHs in biofilm formation is unclear as yet. A recent study has shown that *A. baumannii* ADH may be involved in quorum sensing during biofilm formation, and the inhibition of ADH also reduced the expression of quorum sensing-related signalling [11]. However, it remains unclear whether ADH is driving the quorum sensing process, or merely acting as a feedback modulator; moreover, other functions of ADH during stress resistance and biofilm formation cannot be ruled out at present. Still, the results show that ADHs are critical to *A. baumannii* survival under oxidative stress conditions or in the presence of alcohol substrates, and it is possible that ADHs can serve as targets for the development of novel antiseptic or antibiotic strategies.

Previous research has shown that *A. baumannii* ATCC 17978 cells cultured in ethanol-containing medium subsequently demonstrated increased virulence against *G. mellonella* larvae [15], and the authors speculated that this virulence may be driven by increased production of indole acetic acid (IAA) during ethanol exposure by *A. baumannii*. If this is confirmed, it would be quite concerning, as ethanol-based disinfectants are routinely used in clinical settings. However, it is possible that this virulence may in fact be driven by increased *Adh* gene expression in response to oxidative stress induced by peroxide-based disinfectants or bleach, which are more widely applied in hospital environments and also persist longer on surfaces than volatile ethanol. Previous studies have found that the *AdhE* gene, which encodes a dual-function acetaldehyde-CoA and alcohol dehydrogenase, was upregulated in *Streptococcus pneumoniae* cultured in the presence of ethanol and subjected to oxidative stress, and this resulted in increased virulence against murine RAW 264.7 cells [12]; interestingly, Δ*AdhE* mutant strains demonstrated reduced pathogenicity and virulence [12], and this was also observed in *E. coli* O157:H7 [30]. In this study, we found that *Adh3* and *Adh6* were upregulated in response to oxidative stress (Figure 6 and Figure 7), and Δ*Adh3* and Δ*Adh6* mutant strains exhibited less virulence against *G. mellonella* larvae (Figure 9). Further research to elucidate the underlying mechanisms, as well as field studies to assess *Adh3* and *Adh6* expression in *A. baumannii* samples collected from clinical settings, could help to validate this association.

In conclusion, this study shows that FeADHs have diverse functions in *A. baumannii* beyond ethanol metabolism, being involved in oxidative stress responses and virulence, but different ADHs have varying primary functions. Further research into the additional functions of FeADHs in *A. baumannii*, as well as the roles of other ADHs, is warranted.

## 4. Materials and Methods

### 4.1. Bacterial Strains, Plasmids, and Primers

*A. baumannii* ATCC 19606 and *Escherichia coli* strains were cultured in LB medium at 37 °C with shaking, and solid cultures were grown on LB medium containing 1.5% agar. Mutant *A. baumannii* strains were grown in M9 media (33.7 mM Na_2_HPO_4_, 22 mM KH_2_PO_4_, 8.55 mM NaCl, 9.35 mM NH_4_Cl, 1 mM MgSO_4_, 0.3 mM CaCl_2_), a minimal microbial growth media to which 1% ethanol, 1% 1-propanol, 1% a-butanol, or 5 mM citrate (5 mM citrate medium) was added in accordance with experimental needs. The bacterial strains and plasmids used are presented in Table 3. The primers used in this study are presented in Table 2.

### 4.2. Marker-Less Mutation

Marker-less mutation was performed as previously described [31], with some minor modifications. Briefly, the adjacent regions of the *Adh* gene(s) intended for mutation were cloned into plasmid pk18*mobsacB*, and transformed into *E. coli* S17λπ to generate a donor strain for conjugation with *A. baumannii*. The *E. coli* donor strains and *A. baumannii* recipient strains were cultured in LB medium at 37 °C with shaking for 12 to 16 h, after which a 200 μL aliquot of donor bacterial cells was mixed with recipient *A. baumannii* cells at a 1:20 ratio. The mixed cells were spun down and washed with 60 μL of conjugation buffer (1% NaCl, 10 mM MgSO_4_) to remove traces of LB medium, and the cell pellet was resuspended in 60 μL of conjugation buffer, then spotted onto a membrane filter (47 mm diameter, mixed cellulose esters, A020H047A; Advantech MFS, Dublin, CA, USA) placed on top of 1.5% LB agar. After cultivation at 37 °C for 19 h, filters were washed with conjugation buffer to remove bacterial cells, which were spun down and resuspended in 200 μL of conjugation buffer, then plated to 1.5% LB agar plates containing 50 μg/mL of ampicillin and 50 μg/mL of kanamycin. The first homologous recombination event enables the *E. coli* donor plasmid, which contains a kanamycin-resistant gene, to be integrated into the bacterial chromosome of *A. baumannii* recipient cells. Successful recombinants were then cultivated in LB medium containing 20% sucrose but without kanamycin, thus inducing the *A. baumannii* recombinants to excise the *sacB* gene in a second crossover event, and thereby enabling the deletion of the target *Adh* gene(s). Deletion mutants were subsequently confirmed through PCR analysis.

### 4.3. Recombinant Alcohol Dehydrogenase Purification and Enzyme Activity Assay

Protein overexpression and purification methods were adapted from our previous study [31]. Briefly, putative *A. baumannii Adh* genes were cloned into plasmid pQE80L (Qiagen, Hilden, Germany), which was then transformed into *E. coli* DH5α for recombinant protein production. Ni-affinity chromatography was used for recombinant protein purification. Columns were charged with 1× charge buffer (5 mM NiSO_4_) for 50 min to enable Ni^2+^ binding with the column, after which columns were washed with 1× binding buffer (5 mM imidazole, 0.5 M NaCl, 20 mM Tris-HCl, pH = 7.9) for 50 min. Supernatants containing recombinant proteins were then passed through the column, after which the column was washed with 1× wash buffer (60 mM imidazole, 0.5 M NaCl, 20 mM Tris-HCl, pH = 7.9), and then bound proteins were eluted using 1X elute buffer (1 M imidazole, 0.5 M NaCl, 20 mM Tris-HCl, pH = 7.9). The soluble fraction was collected and passed through Amicon Ultra-15 Centrifugal Filter-10 kDa units (Merck Millipore, Burlington MA, USA) to eliminate proteins smaller than 10 kDA. Retained proteins were concentrated by adding 4× ADH storage buffer (320 mM Tris-HCl, pH = 7.4, 160 mM KCl) to a concentration of 1× and then centrifuging for 20 min at 4 °C, 4000× *g*, after which the supernatant was discarded and 1× ADH storage buffer was added, followed by centrifugation for 10 min at 4 °C, 4000× *g*. Concentrated proteins were divided into aliquots of 250 μL, to which 250 μL of 4×ADH storage buffer and 500 μL of 100% glycerol were added, and then divided into 200 μL aliquots for storage at −80 °C. Purified proteins were used in enzymatic assays as previously described [35]. The enzymatic assay reaction contained a final concentration of 80 mM buffer, 2 mM NAD^+^, 1 mM semicarbazide, and 100 µL of alcohol (ethanol, 1-propanol, or 1-butanol) in 1 mL. The OD_340_ for different protein concentrations was determined every 30 s for three minutes. Specific activity was determined according to the Beer-Lambert Law: A_λ_ = ε_λ_ × C × l, with A_λ_ representing the OD_340_ difference between the third and the first minutes; ε_λ_ = 6.22 mM^−1^cm^−1^, the coefficient of OD_340_; and C indicating the concentration of produced NADH. The production of 1 µmole of NADH was defined as 1 unit of ADH activity [36]. Three buffers with different pH conditions were used: Phosphate buffer (PB buffer; 80 mM Na_2_HPO_4_, 80 mM NaH_2_PO_4_, pH = 5.8–8), Tris-Cl (80 mM Tris-HCl, pH = 8–9.5) and carbonate-bicarbonate buffer (CB buffer; 80 mM Na_2_CO_3_, 80 mM NaHCO_3_, pH = 9.5–10.1).

### 4.4. Stress Resistance Assays

Single colonies of each strain were inoculated in 3 mL LB medium with corresponding antibiotics, which were cultured for 12–16 h at 37 °C with agitation. The overnight culture was refreshed with fresh medium to an OD_600_ of 0.1. Bacteria were treated with 5 mM H_2_O_2_ or 300 µM *tert-*butyl hydroperoxide (*tert*-BHP) for 20 min and 4% NaCl, and viable counts were then determined by dropping 5 µL of cultured medium on LB agar plates 6 times and calculating colony-forming units (CFU) after colonies formed on plates [37].

### 4.5. RNA Extraction and qRT-PCR

Bacterial cultures at an OD_600_ of 0.6 were collected and centrifuged, and pellets were treated with 0.1 volume of fix solution (5% acid/phenol, 95% ethanol), then centrifuged at 4 °C at 12,000× *g* for 10 min. Bacterial pellets were kept at −80 °C before RNA extraction. For RNA extraction, each pellet was suspended in 2 mL of Nucleozol (REF 740404.200, Macherey-Nagel, Düren, Germany) and processed according to the instruction manual provided by the manufacturer. The concentration of RNA was determined using a NanoDrop 2000c Spectrophotometer (Thermo Fisher Scientific, Waltham, MA, USA). DNA contamination was assessed by conducting PCR analysis on a 2 μL RNA sample, using DNA polymerase in the absence of reverse transcriptase. No PCR products were noted, indicating the absence of DNA in purified RNA samples. Following extraction, 2 µg of RNA from each sample was prepared for reverse transcription with 1× RTase buffer, 0.5 mM dNTP, 5 mM DTT, and 200 U of RTase (GScript RTase, MB305-0050, GeneDireX, Taoyuan, Taiwan), and placed at 50 °C for one hour. Quantitative RT-PCR was performed with 100 ng cDNA for each reaction. SYBR Green (PowerSYBR Green PCR Master Mix, cat no. 4367659, Thermo Fisher Scientific) was mixed with cDNA and the corresponding primers (see Table 2) for PCR in a LightCycler^®^ 480 (Roche, Basel, Switzerland) [31].

To ascertain if *Adh* genes contribute to NADH/NAD^+^ homeostasis, the Peredox cassette from pRestB-His7tag-peredox-mCherry (Addgene, Watertown MA, USA), a fluorescent biosensor of the cytosolic NADH-NAD^+^ redox state [33,34], was inserted at an *Eco*RI restriction site into the pWH1266 shuttle vector of *A. baumannii* and *E. coli*, to form pWH1266-pereodox-mCherry. This plasmid was then transferred by electroporation at 1.8 kV, 200 W, 25 μF (MicroPulser; Bio-Rad, Hercules, CA, USA) into wild-type and mutant bacterial strains [32] to measure cytosolic NADH:NAD^+^ ratios. Following incubation at 37 °C for one hour, 200 μL of bacterial culture was spotted onto 1.5% LB agar plates containing 6.25 μg/mL of tetracycline, and plates were subsequently cultured at 37 °C for 12 to 16 h to select for successful transformants.

### 4.6. Fluorescence Analysis

Successful transformants harboring the pWH1266_peredox-mCherry plasmid were cultured in LB medium containing 6.25 μg/mL of tetracycline at 37 °C for 12 to 16 h until OD_600_ = 0.3, after which bacterial cells were concentrated by centrifugation. Cell pellets were resuspended in 300 μL of 0.9% NaCl, from which 50 μL of solution was taken and placed on a slide (1″ × 3″ microscope slides; FEA, Taipei, Taiwan), then mounted with a coverslip (22 mm × 22 mm; Paul Marienfeld, Lauda-Königshofen, Germany) and sealed around the edges using transparent nail polish. Fixatives were not used in this study. The slides were examined under an Eclipse E800 fluorescent microscope (Nikon, Tokyo, Japan), using red (G-2A filter; Ex 510–560/DM 575/BA 590) and green (B-2A filter; Ex 450–490/DM 505/BA 520) fluorescence filters, and at least three different views were captured for each sample.

### 4.7. Virulence Assay with G. mellonella

A virulence comparison was carried out with *A. baumannii* ATCC 19606 wild-type and *Adh3, Adh4,* and *Adh6* single, double and triple mutants of each strain. All procedures were performed as previously described, with minor modifications [16]. Ten *G. mellonella* larvae were selected for the same total weight, and were kept in petri dishes without food prior to infection. Larvae were infected with 5 × 10^6^ CFU of each strain. Overnight cultures of each strain were washed twice with PBS (0.137 M NaCl, 2.7 mM KCl, 10 mM Na_2_HPO_4_, 1.8 mM KH_2_PO_4_), then diluted in PBS. Bacteria in 10 µL aliquots were injected into the hemocoel of each larva via the last left proleg by a Hamilton syringe. Infected larvae were incubated at 37 °C and scored for survival (alive/dead) every 24 h. Larvae were also scored for melanization over 96 h, according to a previously described scoring method [38].

### 4.8. Statistical Analysis

Data were analyzed using two-way ANOVA tests, with *p* < 0.05 denoting significance.

## Figures and Tables

**Table 1:** Alcohol substrate specificity of ADH4.

	Carbonate-Bicarbonate (CB) Buffer, pH = 10.1		
	Ethanol	1-Propanol	1-Butanol
*V_max_* (mmole min^−1^)	0.0064 ± 0.0002	0.027 ± 0.0028	0.005 ± 0.001
*K_M_* (mM)	5.11 ± 0.71	206.8 ± 79.61	196.5 ± 91.21
*k_cat_* (s^−1^)	0.2118	0.9003	0.1683
*k_cat_/K_M_* (s^−1^M^−1^)	41.4481	4.3535	0.8565

**Table 2:** Gene-specific primers used in this study.

Name	Sequences (5′–3′)	Function
pK18_*Adh3*upF	CGAGCTCGGTACCCGGGACGCCCTTTAACATGACCAG	Construction of *Adh3* mutant
pK18_*Adh3*downR	AACGACGGCCAGTGCCACAGATGCGCTAAGGGAAAAC	Construction of *Adh3* mutant
pK18_*Adh4*upF	CGAGCTCGGTACCCGGGTGCCCTTCATTATCAATTTCG	Construction of *Adh4* mutant
pK18_*Adh4*downR	AACGACGGCCAGTGCCAGACATCGCTTTGAGTTGCAT	Construction of *Adh4* mutant
pK18_*Adh6*upF	CGAGCTCGGTACCCGGGACGCACATTGGTCAGTTTTG	Construction of *Adh6* mutant
pK18_*Adh6*downR	AACGACGGCCAGTGCCATTGCTGCAACCATAACAGGT	Construction of *Adh6* mutant
*Adh1*_rF	TGTGATTGCCTGTGGTGAAT	qRT-PCR
*Adh1*_rR	ACACCGCCGTAAAGATGACT	qRT-PCR
*Adh2*_rF	GGTCGATTCATGCCGTACTT	qRT-PCR
*Adh2*_rR	TGTGGTAATACCCGCACAAA	qRT-PCR
*Adh3*_rF	TCAGTTACACCTGCCTATTCTTCA	qRT-PCR
*Adh3*_rR	CCCAAAGCCGACAATAACAT	qRT-PCR
*Adh4*_rF	TGCAAGATGAAGGGCTATTT	qRT-PCR
*Adh4*_rR	CACCGCCTAACGACACAATA	qRT-PCR
*Adh5*_rF	GCCAGCAGATAAAGCGGATT	qRT-PCR
*Adh5*_rR	TGTTGCCCCATATACATTACCA	qRT-PCR
*Adh6*_rF	TCTGGTGCACACAACCTACC	qRT-PCR
*Adh6*_rR	TCTAAAATCGCAGCATGTGG	qRT-PCR
*Adh7*_rF	GGCGAAAATATCGCAACAAT	qRT-PCR
*Adh7*_rR	ACCCAAACCACCAATACCAA	qRT-PCR
pRsetB-His7-Peredox-mCherry_F	GCCCTTTCGTCTTCAAGTAATACGACTCACTATAGGG	Construction of peredox plasmid
pRsetB-His7-Peredox-mCherry_R	AGCTGTCAAACATGAGTCACTTGTACAGTTCGTCCA	Construction of peredox plasmid

**Table 3:** Plasmids and bacterial strains used in this study.

Plasmid	Description	Antibiotic Resistance (µg/mL)	Reference/Source
pK18mobsacB	Suicide vector for homologous recombination	Kan50	[31]
pK18Δ*Adh1*	pK18mobsacB contains the upstream and downstream regions of *Adh1*	Kan50	This study
pK18Δ*Adh2*	pK18mobsacB contains the upstream and downstream regions of *Adh2*	Kan50	This study
pK18Δ*Adh3*	pK18mobsacB contains the upstream and downstream regions of *Adh3*	Kan50	This study
pK18*ΔAdh4*	pK18mobsacB contains the upstream and downstream regions of *Adh4*	Kan50	This study
pK18Δ*Adh5*	pK18mobsacB contains the upstream and downstream regions of *Adh5*	Kan50	This study
pK18Δ*Adh6*	pK18mobsacB contains the upstream and downstream regions of *Adh6*	Kan50	This study
pK18Δ*Adh7*	pK18mobsacB contains the upstream and downstream regions of *Adh7*	Kan50	This study
pQE80L	Expression vector with *colE1* origin for His-tag fusion protein purification	Amp50	Qiagen
p*Adh3*	Ap^r^; *Adh3* cloned into *Bam*HI-*Hin*dIII sites of pQE80L	Amp50	This study
p*Adh4*	Ap^r^; *Adh4* cloned into *Bam*HI-*Hin*dIII sites of pQE80L	Amp50	This study
p*Adh6*	Ap^r^; *Adh6* cloned into *Bam*HI-*Sma*I sites of pQE80L	Amp50	This study
pWH1266	Ap^r^; Tc^r^; shuttle vector for *E. coli* and *A. baumannii*	Amp50, Tc12.5	[32]
pRsetB-His7tag- Peredox-mCherry	Ap^r^; fluorescent biosensor of the cytosolic NADH/NAD^+^ redox state by combining a circularly permuted GFP T-Sapphire with a bacterial NADH-binding protein, Rex, and the red fluorescence of a tandemly attached mCherry.	Amp50	[33,34]
pWH1266_*peredox-mCherry*	Ap^r^; Tc^r^; permuted GFP T-Sapphire with a bacterial NADH-binding protein, Rex, and the red fluorescence of a tandemly attached mCherry from pRsetB-His7tag- Peredox-mCherry was cloned into *Eco*RI sites of pWH1266	Amp50, Tc12.5	This study
**Strain**	**Description**		**Reference/Source**
*E. coli* DH5α	F^−^, *supE44*, *hsdR17*, *recA1*, *gyrA96*, *endA1*, *thi-1*, *relA1*, *deoR*, λ^−^		ATCC53868
*Acinetobacter baumannii* ATCC 19606	Primary strain used in this study		[31]
∆*Adh3*(∆*3*)	Marker-less *Adh3* deletion mutant		This study
∆*Adh4*(∆*4*)	Marker-less *Adh4* deletion mutant		This study
∆*Adh6*(∆*6*)	Marker-less *Adh6* deletion mutant		This study
∆*Adh34*(∆*34*)	Marker-less *Adh34* double deletion mutant		This study
∆*Adh36*(∆*36*)	Marker-less *Adh36* double deletion mutant		This study
∆*Adh46*(∆*46*)	Marker-less *Adh46* double deletion mutant		This study
∆*Adh346*(∆*346*)	Marker-less *Adh346* triple deletion mutant		This study

Amp: ampicillin; Kan: kanamycin.

## Data Availability

The data presented in this study are available on request from the corresponding author. The data are not publicly available as the full dataset is undergoing analysis to guide future research and potential publications.

## Supplementary Materials

The following are available online at https://www.mdpi.com/article/10.3390/ijms22189921/s1.
