# Supplementary material for: Role of Iron-Containing Alcohol Dehydrogenases in Acinetobacter baumannii ATCC 19606 Stress Resistance and Virulence
Source: Int J Mol Sci. 2021 Sep 14;22(18):9921. doi: 10.3390/ijms22189921 (PMC8465190; doi:10.3390/ijms22189921)
Supplement: Supplementary file 1 [file ijms-22-09921-s001.zip › ijms-1343472-supplementary.pdf]

Role of iron-containing alcohol dehydrogenases in *Acinetobacter baumannii* ATCC 19606 stress resistance and virulence

Supplementary Material

Table S1. Seven *Adh* genes identified from the GenBank database for *A. baumannii*

Protein	Gene		Gene Size	Type
ADH1	*Adh1*	*DJ41_2111*	1,164 bp	Zinc-containing, GroES-like protein
ADH2	*Adh2*	*DJ41_435*	1,053 bp	Zinc-containing
ADH3	*Adh3*	*DJ41_189*	1,158 bp	Iron-containing
ADH4	*Adh4*	*DJ41_136*	1,173 bp	Iron-containing
ADH5	*Adh5*	*DJ41_1604*	759 bp	Short-chain
ADH6	*Adh6*	*DJ41_3559*	1,185 bp	Iron-containing
ADH7	*Adh7*	*DJ41_3039*	1,029 bp	Zinc-containing

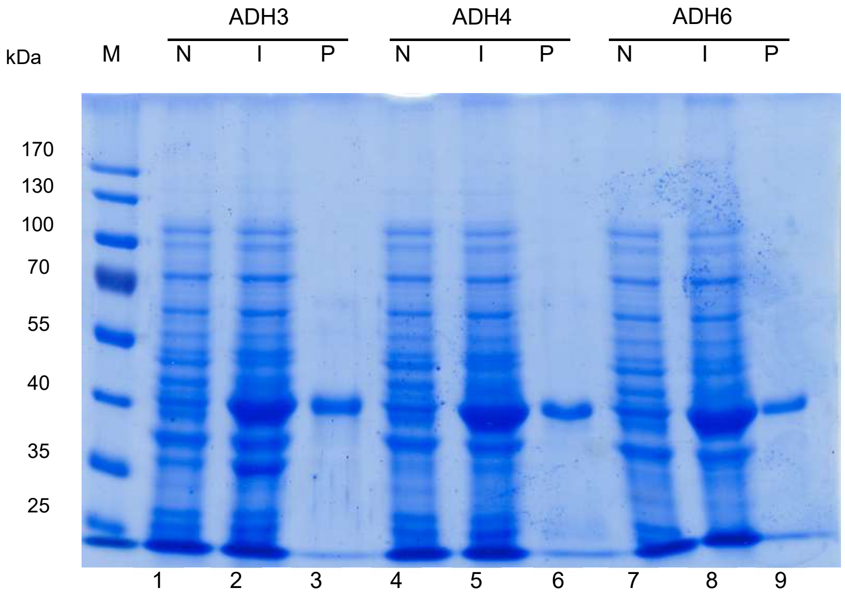

Figure S1. Results of 12% SDS-PAGE analysis of FeADHs (ADH3, ADH4, and ADH6). N represents the non-induction protein fraction and I represents the induction fraction, while P represents purified protein.

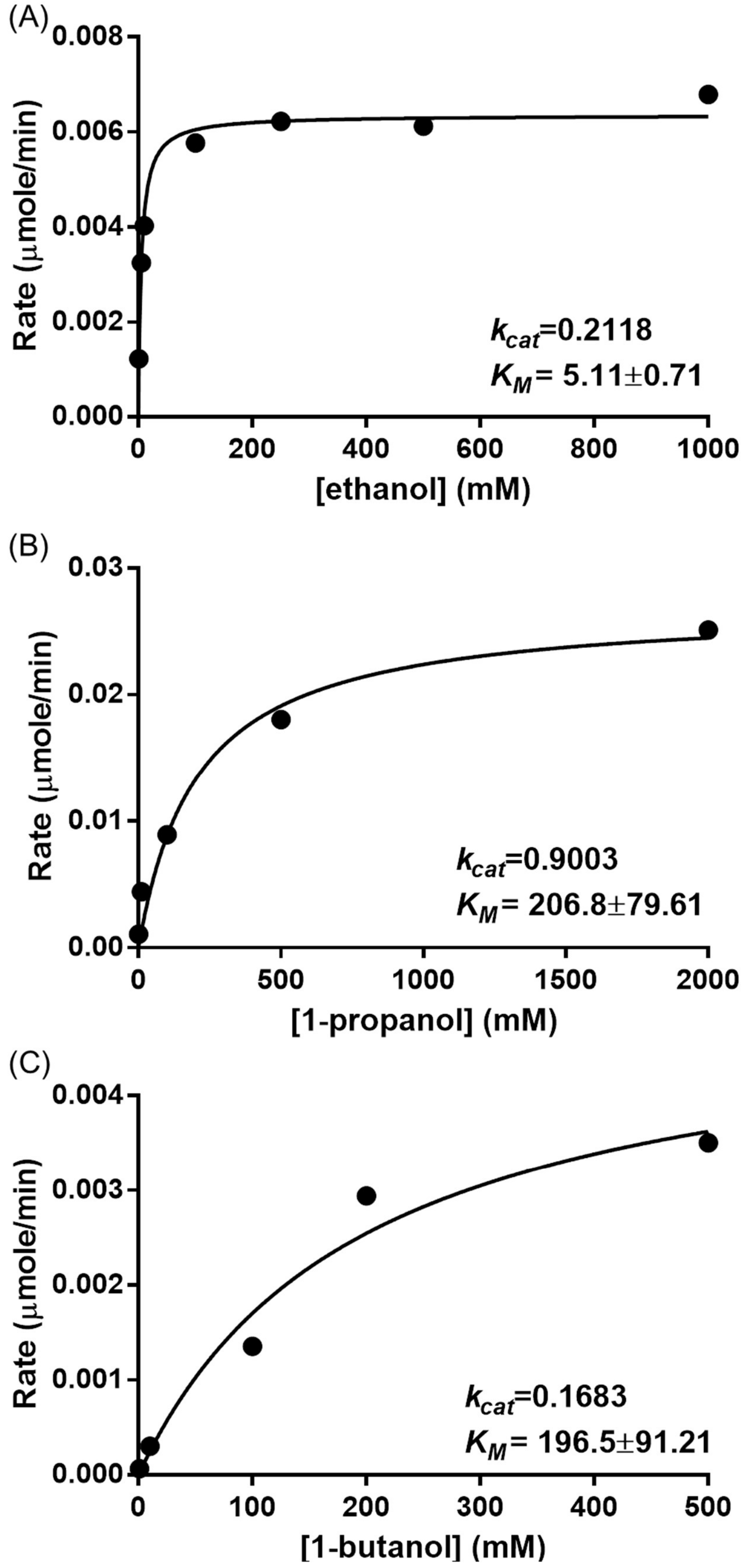

Figure S2. Michaelis-Menten enzyme kinetics of the ADH4 enzyme for different substrates. Enzyme kinetics for (**A**) ethanol; (**B**) 1-propanol; and (**C**) 1-butanol are shown.

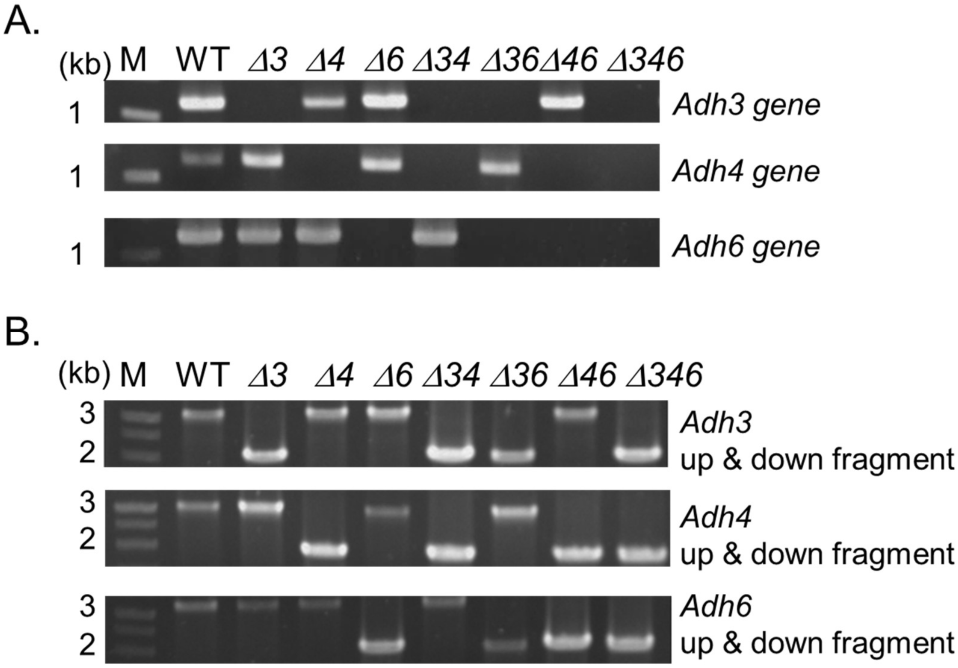

Figure S3. Agarose gel electrophoresis of *Adh* gene colony PCR products for *Adh* mutant identification. (**A**) Confirmed excision of *Adh* gene(s) in mutants. (**B**) Confirmed excision of *Adh* gene(s) and presence of the up and down fragment in mutants.

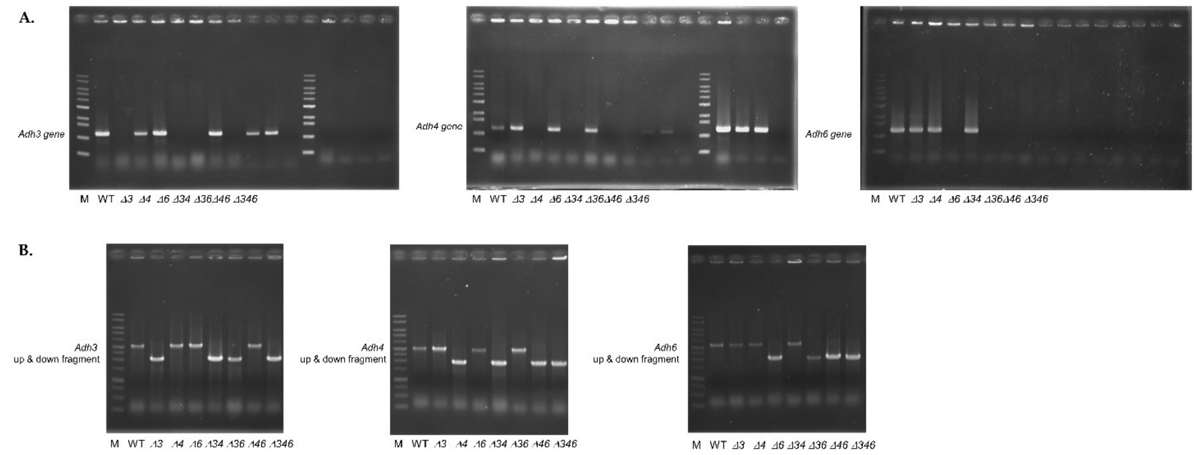

Figure S4. Uncropped agarose gel electrophoresis results of *Adh* gene colony PCR products. (**A**) Confirmed excision of *Adh* gene(s) in mutants. (**B**) Confirmed excision of *Adh* gene(s) and presence of the up and down fragment in mutants.
